# Exploring Functional Polymers in the Synthesis of Luminescent ZnO Quantum Dots for the Detection of Cr^6+^, Fe^2+^, and Cu^2+^

**DOI:** 10.3390/polym16030429

**Published:** 2024-02-03

**Authors:** Leire San José, Nastasiya Yuriychuk, Olga García, Mar López-González, Isabel Quijada-Garrido

**Affiliations:** Group of Nanohybrids and Interactive Polymers, Instituto de Ciencia y Tecnología de Polímeros, Consejo Superior de Investigaciones Científicas (ICTP-CSIC), C/Juan de la Cierva, 3, 28006 Madrid, Spainmar@ictp.csic.es (M.L.-G.)

**Keywords:** ZnO QDs, functionalized polymers, nanohybrids, fluorescent nanomaterials, RAFT polymerization, Cr^6+^ sensing

## Abstract

The main aim of this work is to demonstrate that well-defined methacrylate-based copolymers with oligoethylene glycol side chains and functional groups such as thiol and glycidyl, obtained by photo-initiated reversible addition-fragmentation chain transfer (RAFT) in ethanol, are highly suitable as templates in the synthesis and protection of ZnO quantum dots (ZnO QDs) with remarkable photoluminescent properties. While the affinity of thiol groups to metallic surfaces is well established, their interaction with metal oxides has received less scrutiny. Furthermore, under basic conditions, glycidyl groups could react with hydroxyl groups on the surface of ZnO, representing another strategy for hybrid synthesis. The size and crystalline morphology of the resulting hybrids were assessed using DLS, TEM, and XRD, indicating that both polymers, even with a low proportion of functional groups (5% mol) are appropriate as templates and ligands for ZnO QDs synthesis. Notably, thiol-containing polymers yield hybrids with ZnO featuring excellent quantum yield (up to 52%), while polymers with glycidyl groups require combination with the organosilane aminopropyl triethoxysilane (APTES) to achieve optimal results. In both cases, these hybrids exhibited robust stability in both ethanol and aqueous environments. Beyond fundamental research, due to the remarkable photoluminescent properties and affordability, these hybrid ZnO QDs are expected to have potential applications in biotechnology and green science; in particular, in this study, we examined their use in the detection of environmental contaminants like Fe^2+^, Cr^6+^, and Cu^2+^. Specifically, the limit of detection achieved at 1.13 µM for the highly toxic Cr^6+^ underscores the significant sensing capabilities of the hybrids.

## 1. Introduction

**Scheme 1:**
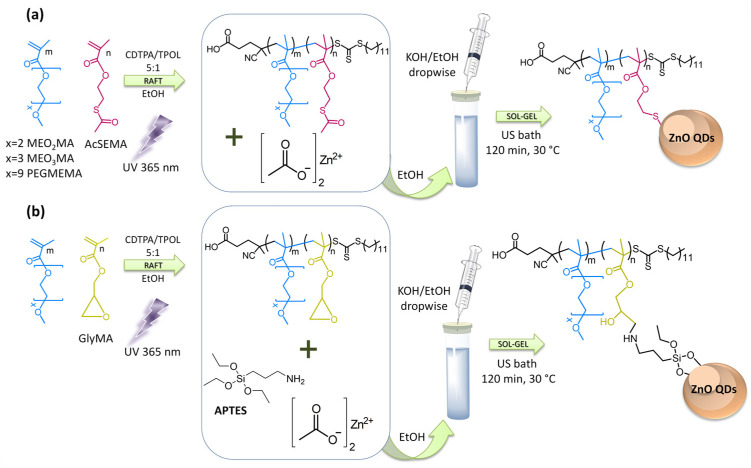
Synthesis of functional statistical copolymers by Photo-RAFT using CDTPA RAFT agent and TPOL photoinitiator in ethanol medium under 365 nm UV light. (**a**) Copolymers with thioacetate groups are used alone as templates for the synthesis of ZnO QDs, whereas (**b**) copolymers with epoxy groups are combined with APTES. Under the basic medium of the reaction, the thioacetate releases a thiol group with avidity for the ZnO QD surface and the epoxy group reacts with the amine of the silane on the ZnO QD surface.

Zinc oxide quantum dots (ZnO QDs) are nanoscale particles of only a few nanometers which possess unique optical and electronic properties that differ from their bulk counterpart. Due to their size-dependent optical properties and several advantages, such as low toxicity and low cost, ZnO QDs are used in a wide range of applications such as bioimaging [1,2], drug delivery [1,3], sensing [1,4,5,6,7], and optoelectronics [8]. Additionally, ZnO QDs possess antimicrobial properties [1,9,10,11], making them useful in applications such as water purification [10], food packaging [11], and medical devices [12]. ZnO QDs can be synthesized using simple and low-cost methods such as the sol-gel method, which is particularly widely favored due to its simplicity. This process typically involves the hydrolysis of zinc acetate in ethanol under sonication, resulting in the formation of nanoparticles ranging from 3 to 5 nm with an emission wavelength of about 500–570 nm. However, one disadvantage of this method is that the as-synthesized particles often lack colloidal stability and tend to undergo Ostwald ripening, which causes them to precipitate in the reaction medium after a short period of time. This leads to a decrease in their optical and electronic properties. To overcome this issue, several strategies can be employed, including the use of stabilizing agents, such as β-cyclodextrin [4,7], organosilanes [13,14], and polymers [14,15,16,17]. These agents can be added together during the ZnO QDs synthesis (in situ) [14] or post-modification [15] to preserve the stability and luminescence of ZnO QDs. However, it is noteworthy that the effect of the polymeric ligand during the synthesis of ZnO QDs has been investigated to a lesser extent than other types of QDs that are less environmentally friendly. In a previous contribution to the topic, homopolymers and amphiphilic block copolymers decorated with hydroxyl functionalities were used as ligands and templates for the stabilization of ZnO QDs [14]. The polymer coating resulted in a significant improvement in the long-term stability, especially when they were combined with organosilanes [14]. In this new contribution, we will explore the use of other functionalities such as thiol and epoxy groups along the macromolecular chain of poly(oligoethylene glycol methacrylate). While the strong interaction between thiol and the surface of noble metal NPs [18,19], Cu nanoclusters (CuNCs) [20], and semiconductor CdSe QDs [21] is well-established, there has been relatively less research on the interaction between metal oxide and thiol. However, some recent reports suggest an affinity between thiols and ZnO surfaces [22,23]. For this reason, in the present work, monomers with a protected thiol group, 2-(acetylthio)ethyl methacrylate (AcSEMA), are copolymerized with methacrylic copolymers with oligoethylene glycol side chains of different lengths by RAFT photo-polymerization, which can be used as templates for the in situ synthesis of ZnO QDs (Scheme 1a). Moreover, since in our previous contribution [14] hydroxylated polymers in combination with aminopropyl triethoxysilane (APTES) resulted in a noticeable enhancement of the long-term luminescence of ZnO QDs, we hypothesized that polymers with epoxy groups, under the basic medium used in the reaction, could form a covalent bond with the amino group of APTES organosilane on the surface of ZnO QDs. Thus, oligo ethylene glycol methacrylates and glycidyl methacrylate (GlyMA) are statistically copolymerized by RAFT, as shown in Scheme 1b, and are also used as templates for the synthesis of ZnO QDs. The research problem addressed in this study pertains to the relatively limited exploration of the impact of polymeric ligands in the synthesis of ZnO quantum dots (QDs) compared to less environmentally friendly QDs. While previous research focused on hydroxyl-functionalized polymers [14], this study introduces thiol and epoxy groups along the poly(oligoethylene glycol methacrylate) chain. The research investigates the influence of binding groups, polymer concentration, and functional group ratios on the photoluminescence of the QDs in ethanolic and aqueous media. Additionally, the study explores the potential of these polymer-functionalized ZnO QDs for metal ion detection, emphasizing their applicability in environmental sensing. This research contributes to advancing knowledge about tunable polymeric ligands in ZnO QD synthesis, enhancing stability, and expanding their applications in sensing.

## 2. Materials and Methods

### 2.1. Materials

Poly(ethylene glycol) methyl ether methacrylate (PEGMEMA) (Sigma-Aldrich, Darmstadt, Germany), average M_n_~500), di(ethylene glycol) methyl ether methacrylate (MEO_2_MA) (Sigma-Aldrich, 95%), triethylene glycol methyl ether methacrylate (MEO_3_MA) (Sigma-Aldrich, 94%), glycidyl methacrylate (GlyMA) (Fluka, Buchs, Switzerland, ≥97%), (4-cyano-4-[(dodecylsulfanylthiocarbonyl) sulfanyl]pentanoic acid (CDTPA) (Sigma-Aldrich, 97%), ethyl (2,4,6-trimethylbenzoyl)phenylphosphinate (TPOL) (Fluorochem, Hadfield, UK, 95%) were used for polymer synthesis. The monomer with the protected thiol group, 2-(acetylthio)ethyl methacrylate (AcSEMA), was synthesized according to the protocol previously described by our team [21]. Zinc acetate dihydrate Zn(CH_3_COO)_2_·2H_2_O (Sigma-Aldrich, ≥99%), potassium hydroxide (KOH) (Panreac, Barcelona, Spain, 85%), (3-aminopropyl)triethoxysilane (APTES) (Sigma-Aldrich, ≥99%) were used for ZnO QDs’ synthesis. Rhodamine 6G for quantum yield (Φ_F_) determination was provided by Luxottica-Exciton (Lockbourne-OH, USA).

Metallic salts for quenching studies include: lithium bromide (LiBr, Honeywell, Charlotte, NC, USA, ≥99%), magnesium oxide (MgO, Sigma-Aldrich, >97%), potassium carbonate (K_2_CO_3_, Panreac, ≥99.9%), calcium hydride (CaH_2_, Sigma-Aldrich, >95%), potassium chromate (K_2_CrO_4_, Sigma-Aldrich, >99%), manganese(II) sulfate monohydrate, (MnSO_4_·H_2_O, Sigma-Aldrich, >99%), iron(II) chloride tetrahydrate (FeCl_2_·4H_2_O, Aldrich, Darmstadt, Germany, ≥99%), iron(III) chloride hexahydrate (FeCl_3_·6H_2_O, Aldrich, ≥99.9%), cobalt nitrate hexahydrate (Co(NO_3_)_2_·6H_2_O, Panreac, ≥99.9%), nickel nitrate hexahydrate (Ni(NO_3_)_2_·6H_2_O, Fluka, ≥98.5%), copper(II) perchlorate hexahydrate (Cu(ClO_4_)_2_·6H_2_O, Aldrich, ≥98%), cadmium perchlorate hydrate (Cd(ClO_4_)_2_·H_2_O, Aldrich, ≥99.9%), mercury(II) chloride (HgCl_2_, Panreac, ≥99.9%), and lead(II) nitrate (Pb(NO_3_)_2_, Aldrich, ≥99%). which were all used as received. Absolute ethanol (Merck, Darmstadt, Germany, 99.8%), methanol (Merck, ≥99.9%), Milli-Q water, hexane (Scharlau, Barcelona, Spain, HPLC grade). All reagents were used as received without further purification.

### 2.2. Synthesis of Copolymers via RAFT Photo-Polymerization

PEGMEMA, MEO_2_MA, and MEO_3_MA monomers, from now on denoted as EG, DEG, and TEG respectively, were combined along with AcSEMA (AcS) or GlyMA (GM) monomers in order to synthesize statistical copolymers by RAFT photopolymerization (Scheme 1). These polymers were further used as templates and ligands for the ZnO QDs synthesis (Scheme 1).

The photopolymerizations were carried out in a UVP CL-1000 Ultraviolet Crosslinker photoreactor under 4 lamps irradiating UV light centered at 365 nm for 2 h, producing a power of 29 W m^−2^, experimentally determined with a spectroradiometer (v2.2, LuzChem, Ottawa, ON, Canada). The typical protocol for the synthesis of the copolymers is described below for the sample EG_95_-AcS_5_: PEGMEMA (0.9943 g, 1.93 mmol), AcSEMA (0.0191 g, 0.1 mmol), and CDTPA RAFT agent (0.0164 g, 0.041 mmol) were added to a glass reaction tube with 1.9 mL of absolute ethanol (1.5 g, 32.6 mmol) followed by the addition of TPOL photoinitiator (2.57 mg, 0.0081 mmol, CDTPA/TPOL ratio = 5). This resulted in a yellow solution that was purged with nitrogen for 20 min in an iced-water bath followed by another 10 min at room temperature. The sealed tube was placed inside the photoreactor and it was continuously irradiated and magnetically stirred for 2 h. After that, the reaction was exposed to air and immersed in an iced-water bath. The raw reaction was diluted in methanol and precipitated in hexane twice in order to remove the unreacted precursors. Purified polymers were high vacuum dried and stored at 4 °C. The estimated monomer conversion, according to ^1^H-NMR spectra, is shown in Table 1.

In order to evaluate the influence of the ratio of binding group (thiol or epoxy), copolymers of different functional monomeric contents were synthesized as shown in Table 1, fixing the monomer/CDTPA RAFT agent molar ratio to 17, 25, or 50.

### 2.3. Synthesis of Fluorescent ZnO QDs

The synthetic procedure for the ZnO QDs synthesis, based on the sol-gel method, starts preparing the organometallic precursor, 25 mL of 0.06 M solution of zinc acetate dihydrate (Zn(CH_3_COO)_2_·2H_2_O), in absolute ethanol. The solution is refluxed at 80 °C in a round bottom flask and magnetically stirred for 1 h. The typical protocol for the synthesis is described below for the sample ZnO@EG_95_-AcS_5_-2: after cooling to room temperature, 5 mL (0.3 mmol) of the zinc acetate solution is taken and transferred to a glass reaction tube, followed by the addition of 19.4 mg of EG_95_-AcS_5_ (which corresponds to 2 µmol of AcSEMA functional monomer, indicated on the label) dissolved in 2.48 mL of ethanol. The tube is then sealed and placed inside an ultrasonic bath at 30 °C (frequency of 37 kHz), where 0.66 mL of KOH 0.9 M solution is added dropwise in a OH^−^/Zn^2+^ molar ratio of 2:1. The obtained clear solution with the coated ZnO QDs is cooled down in an iced-water bath and stored at 4 °C protected from light. For XRD and FTIR analysis, samples were concentrated in ethanol and precipitated in hexane to remove the excess of precursors.

### 2.4. Characterization and Properties

^1^H-NMR and ^13^C-NMR spectra were recorded in CDCl_3_ using a Bruker Avance III-HD-400 spectrometer (Bruker, Madrid, Spain). Molecular weight distributions and dispersity (*Ð* = M_w_/M_n_) of copolymers were determined by SEC using DMF stabilized with 0.1 wt% of LiBr as the mobile phase at a flow rate of 0.7 mL min^−1^ and at 50 °C on a Waters 1525 Binary HPLC with a refractive index detector (Waters 2414). Calibration was performed using poly(methyl methacrylate) standards. Infrared spectra were recorded using a Perkin-Elmer Spectrum Two FTIR spectrometer fitted with an Attenuated Total Reflectance (ATR) accessory. XRD was employed to analyze the crystalline structure of the ZnO QDs. Diffractograms were recorded in the reflection mode by using a Bruker D8 Advance diffractometer provided with a PSD Vantec detector (from Bruker, Madrid, Spain). CuKα radiation (λ = 0.1542 nm), operating at 40 kV and 40 mA. The equipment was calibrated with different standards. The diffraction scans were collected within the range of 2*θ* = 4–80°, with a 2*θ* step of 0.024° and 0.5 s per step. ZnO QDs morphology and size was determined by TEM in a JEOL JEM-2100 HT microscope operated at 200 kV and equipped with a LaB6 gun, an CCD ORIUS SC1000 (Model 882) camera, STEM unit with ADF detector, and a point resolution of 0.25 nm. This microscope is located at ICTS Centro Nacional de Microscopía Electrónica at UCM (Madrid, Spain). Mean particle size was obtained by measuring at least 150 particles. The hydrodynamic size of the nanoparticles was determined by DLS. Diluted samples in ethanol were measured at 20 °C to determine the hydrodynamic size as the number distribution by a Zetasizer Nano ZS instrument (Malvern Instruments Ltd., Madrid, Spain). Malvern Zetasizer Software 7.12 was used for data acquisition and analysis. The emission spectra of hybrid ZnO QDs were recorded on a Perkin Elmer FL6500 spectrophotometer (Perkin-Elmer, Madrid, Spain) in ethanol at the excitation wavelength of 365 nm, using a Rhodamine 6G solution in ethanol as standard. The absorption spectra of the synthesized ZnO QDs and Rhodamine 6G in ethanol, were recorded on a UV/Vis NanoDrop One Thermo-Scientific spectrometer. Quantum yield (Φ_F_) of the ZnO QDs, was calculated, as in a previous work [14], by the relative method using Rhodamine 6G as standard.

Experiments to investigate the use of hybrid ZnO QDs as sensors for metal detection were carried out by incubating during 30 min, 50 μL of the sample reaction diluted 1:10 in water, with 50 μL of different salts solutions to obtain a final concentration between 5 and 100 μM, and studying the resulting fluorescence emission signal.

## 3. Results and Discussion

### 3.1. Synthesis of Functional Copolymers

**Figure 1:**
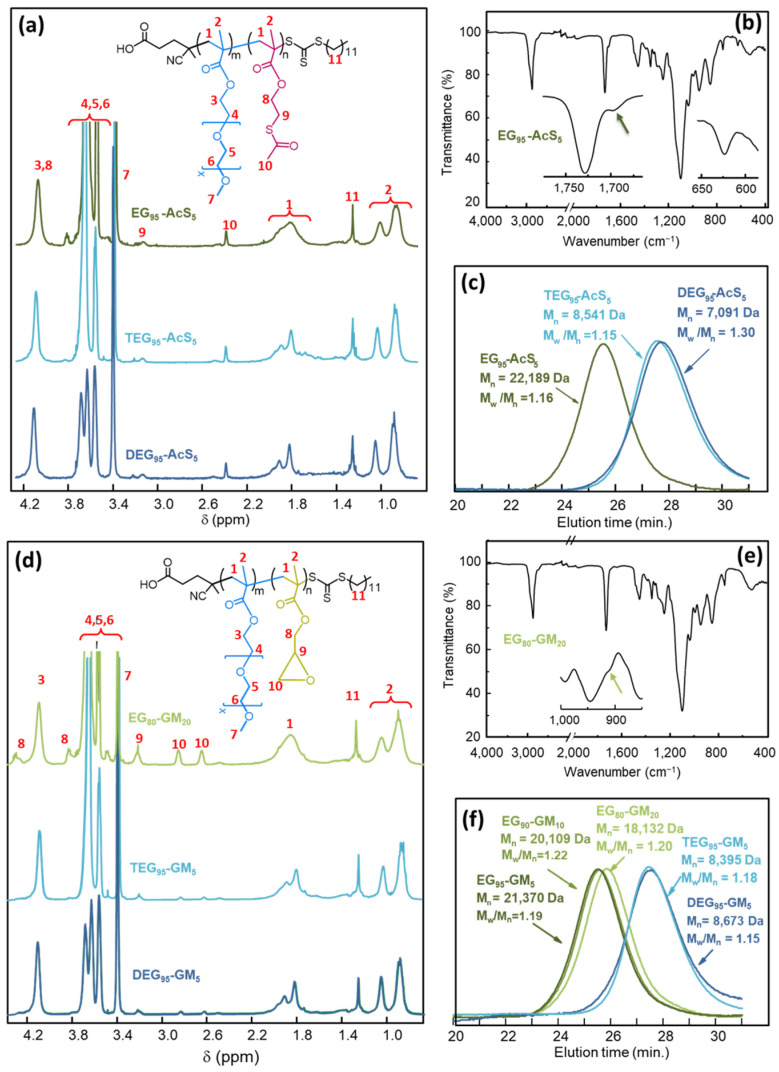
(**a**) ^1^H-NMR (**b**) FTIR and (**c**) SEC traces, corresponding to representative copolymers of oligoethylene glycol methacrylate (PEGMEMA = EG, MEO_3_MA = TEG, or MEO_2_MA = DEG) and AcSEMA (AcS). (**d**) ^1^H-NMR, (**e**) FTIR, and (**f**) SEC traces, corresponding to representative copolymers of oligoethylene glycol methacrylate (PEGMEMA = EG, MEO_3_MA = TEG, or MEO_2_MA = DEG) and GlyMA (GM).

For the synthesis of the thiolated copolymers, AcSEMA, which features a protected thiol group, has been synthesized in our laboratory. In previous years, well-defined copolymers with AcSEMA were synthesized by our group using atom transfer radical polymerization (ATRP) [18,21] for the fabrication of different nanohybrids. The protected thiol group allows for the use of free radical and controlled radical polymerization techniques such as RAFT. In fact, RAFT has also been successful in synthesizing these types of copolymers with applications in the preparation of gold nanoparticle-based luminescent nanohybrids [19]. The RAFT polymerization process was activated using a TPOL photoinitiator and a 365 nm UV light (Scheme S1). The polymers have been successfully synthesized in ethanol, which confers an added advantage as ethanol also serves as the medium for the synthesis of ZnO QDs. In Figure 1a,b, ^1^H-NMR spectra and FTIR, respectively, corresponding to representative AcSEMA copolymers are displayed. The presence of proton signals at 3.13 ppm (related to CH_2_S) and 2.39 ppm (associated with CH_3_CO) in Figure 1a, along with the observed shoulder at 1695 cm^−1^ and the band at 622 cm^−1^ (C=O stretching and C(O)-S stretching, respectively) in the FTIR spectrum (Figure 1b), supports the presence of the AcSEMA moieties into the copolymers. In Figure 1d,e, ^1^H-NMR spectra and FTIR corresponding to representative GlyMA copolymers are displayed. The proton signals at 4.26, 3.21, 2.83, and 2.62 ppm (Figure 1d) and the shoulder at 905 cm^−1^ (Figure 1e), corresponding to the ring vibration in the oxirane group, provide confirmation of the accurate introduction of GlyMA into the copolymer structure. As can be seen in Table 1, the experimental AcSEMA and GlyMA molar values calculated from ^1^H-NMR, are in good agreement with the feed composition. The SEC traces displayed in Figure 1c,f for AcSEMA and GlyMA copolymers reveal a narrow distribution of polymer chains. The *M*_n_ values are close to the theoretical ones; in addition, polymers present dispersity values of about 1.2 in all cases, indicating a good control of the RAFT processes.

### 3.2. Synthesis of Hybrid ZnO QDs Using Functional Copolymers as Templates

**Figure 2:**
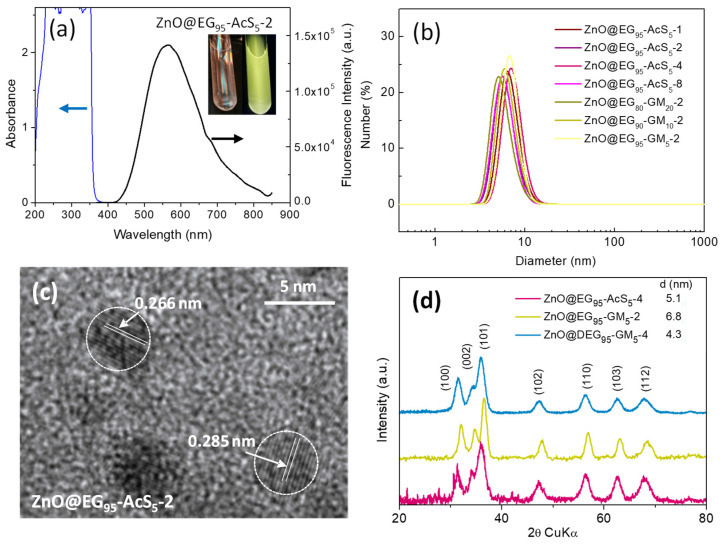
(**a**) Absorption and emission spectra (after excitation at 365 nm) corresponding to a sample synthesized in the presence of thiolated polymer (EG_95_-AcS_5_-2); (**b**) hydrodynamic diameter determined by Number (%) corresponding to ZnO QDs functionalized with copolymers bearing thiol or glycidyl groups; (**c**) TEM image corresponding to the ZnO@EG_95_-AcS_5_-2 hybrid, the distance between the lattice planes are indicated in the Figure; (**d**) XRD of some representative ZnO@polymer hybrids.

For the synthesis of the ZnO@polymer hybrids, a solution of zinc acetate in ethanol was hydrolyzed by adding KOH in the presence of polymers with either thiol or epoxy groups, while subjecting the solution to an ultrasonic bath, as illustrated in Scheme 1. After 120 min of reaction, samples are transparent under ambient light and luminescent under UV light (inset in Figure 2a). All the hybrids exhibit strong absorption below 350 nm and green-yellow emissions centered at about 550–560 nm (Figure 2a). The DLS measurements depicted in Figure 2b clearly demonstrate the formation of ZnO QDs with hydrodynamic diameters below 10 nm when RAFT polymer ligands are present. The hydrodynamic diameters obtained from the Number distribution of the dynamic light scattering (DLS) measurements are compiled in Table 2 and Table 3. The hybrid hydrodynamic diameters range from 5.5 to 7.8 nm, showing no discernible trend based on the composition of the polymer ligand or the molecular weight of the polymer. The crystalline structure of the ZnO QDs is predominantly wurtzite, as indicated by the parallel lines in Figure 2c representing the d-spacing of the (002) (0.26 nm) and (100) (0.28 nm) planes, while the dashed-line circles enclose the nanoparticles. The XRD patterns of representative samples, as depicted in Figure 2d, exhibit characteristic patterns consistent with wurtzite crystal structure (JCPDS file No. 80-0075). The peak broadening observed is attributed to the nanoscale size of the particles, and, based on the diffraction peaks at 2*θ* = 56.7 and 47.4, the nanoparticle size can be estimated to range from 4.3 to 6.8 nm, as indicated in Figure 2d.

The surface of the Zn QDs can be effectively silanized using alkoxysilanes [14,24,25], presenting a robust approach for maintaining luminescence in both organic and aqueous environments. In a previous study [14], we successfully demonstrated that combining silanes with hydroxylated polymers was a favorable strategy to achieve long-term luminescence in ZnO QDs. Building upon these findings, the present work aims to achieve the durable covalent functionalization of the ZnO surface. For this purpose, we employed oligoethylene glycol polymers with epoxy groups in conjunction with APTES, a silane compound containing amino groups. The basic nature of the reaction medium facilitates ring opening in the epoxy groups, enabling the formation of covalent bonds between the epoxy groups and APTES; whereas the silanol group, after the hydrolysis of the silane and condensation on the surface of ZnO QDs, leads to a good protective coating. These ZnO QDs functionalized with APTES and copolymers exhibit a similar size and crystalline structure (Table 2) to those without APTES; however, they differ in terms of their optical properties, as will be discussed later.

**Figure 3:**
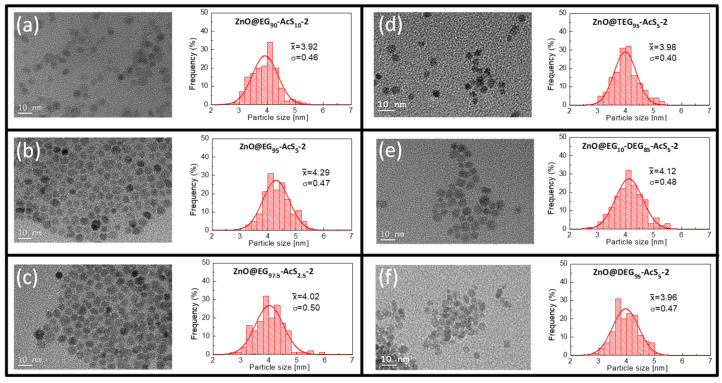
Representative TEM images of ZnO QDs with AcSEMA copolymer coating: (**a**) ZnO@EG_90_-AcS_10_-2; (**b**) ZnO@EG_95_-AcS_5_-2; (**c**) ZnO@EG_97.5_-AcS_2.5_-2; (**d**) ZnO@TEG_95_-AcS_5_-2; (**e**) ZnO@EG_10_-DEG_85_-AcS_5_-2; (**f**) ZnO@DEG_95_-AcS_5_-2. Insert show particle size histograms corresponding to the samples indicated in the Figure.

Figure 3 displays the Transmission Electron Microscopy (TEM) images of the hybrids containing copolymers with AcSEMA. These images reveal interesting insights into the morphology and structure of the nanoparticles. Remarkably, in all cases examined, the nanoparticles showcase a remarkably uniform spherical form, with an average size of approximately 4 nm. Furthermore, their distribution across the samples appears to be well-dispersed, which is indicative of a controlled synthesis process. Moreover, our investigation extends to copolymers synthesized in the presence of epoxy groups and a combination of epoxy-APTES, as depicted in Figure S1. Encouragingly, these copolymers also demonstrate a similar pattern, exhibiting nanoparticles with a spherical shape and a comparable size range of around 4 nm. The ability to consistently achieve such well-defined nanostructures with distinct functionalities evidences the versatility and precision of the synthesis method.

**Figure 4:**
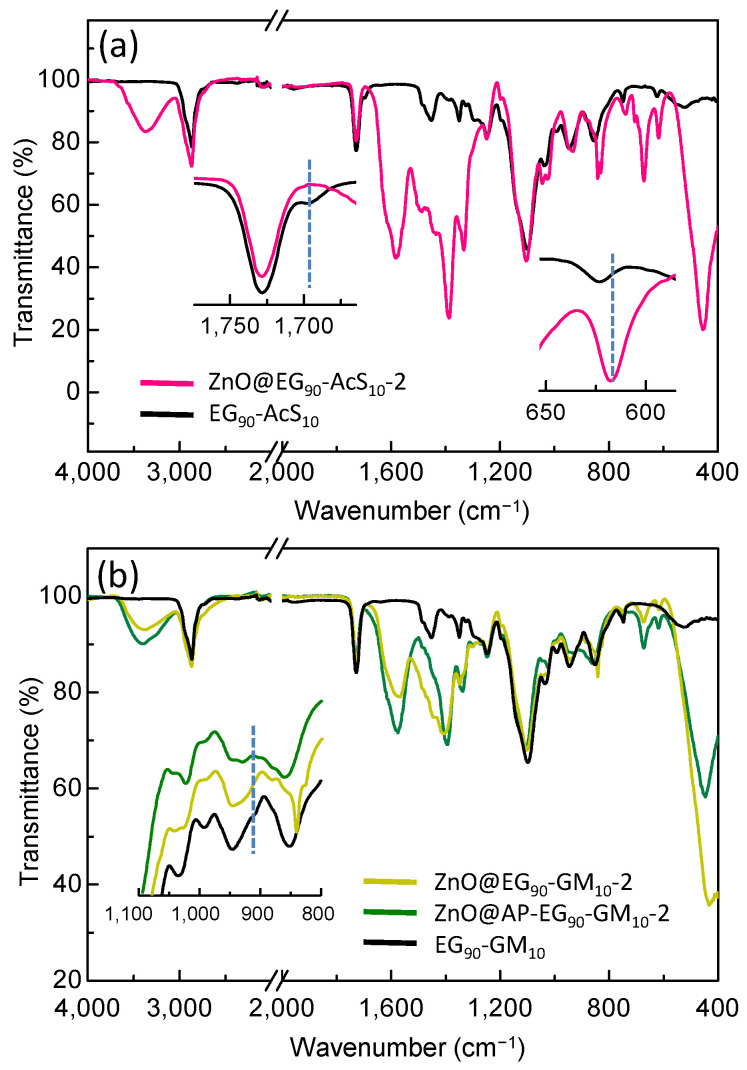
Comparative ATR-FTIR spectra analysis between (**a**) ZnO@-EG_90_-AcS_10_-2 QDs and the pristine EG_90_-AcS_10_ copolymer, and (**b**) ATR-FTIR spectra corresponding to ZnO@EG_90_-GM_10_-2 and ZnO@AP-EG_90_-GM_10_-2 QDs compared to the pristine EG_90_-GM_10_ copolymer.

Figure 4 illustrates the FTIR spectra of ZnO QDs with thiol or epoxy polymer templates, along with the spectra of the pristine copolymers, providing valuable insights into their chemical properties. The spectra corresponding to ZnO@polymer exhibit distinct bands at approximately 460–440 cm^−1^, attributed to Zn-O. Moreover, the presence of oligoethylene glycol polymers is evidenced by the observed vibration modes, the strong stretching vibration in the -C=O group at 1725 cm^−1^ and at approximately 1090 cm^−1^, and the typical C-O-C stretching in ether, confirming successful functionalization. Additionally, residual potassium acetate is indicated by bands at 1560 and 1400 cm^−1^ in ZnO QDs with polymer ligands. For the successful capping of the ZnO QDs, a crucial aspect is the release of the functional group with avidity for the ZnO surface. This can be achieved by converting the thioacetate group in AcSEMA into a thiol in the presence of KOH. The effectiveness of this conversion is evident from the changes observed in the FTIR spectrum (Figure 4a). Specifically, the disappearance of the shoulder at around 1696 cm^−1^ and the band at 623 cm^−1^, corresponding to thioacetate C=O stretching and C(O)-S stretching, respectively, indicates the successful transformation of thioacetate into thiol. Moreover, the opening of the epoxide ring is suggested by the disappearance of the shoulder corresponding to the glycidyl group around 906 cm^−1^ (Figure 4b). In the presence of APTES, additional absorptions attributed to Si-O-Si are observed (Figure 4b).

**Figure 5:**
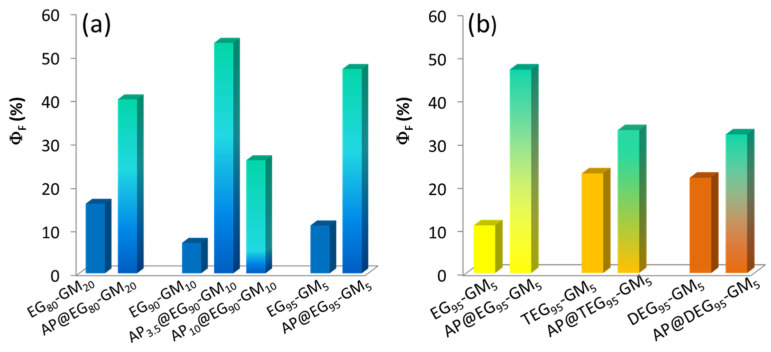
Fluorescence Quantum Yield (Φ_F_) of ZnO QDs in ethanol (**a**) comparing the use of copolymers of PEGMEMA (EG) with glycidyl groups with and without APTES; (**b**) comparing EG, DEG, and TEG in copolymers with glycidyl groups.

The Φ_F_ of ZnO QDs, using copolymers of PEGMEMA (EG) with glycidyl groups as templates, has been found to exhibit low values, ranging between 0.05 and 0.16 (Table 2 and Figure 5a), when compared with previous results [14]. However, there are interesting results when utilizing oligoethylene glycols with short chain lengths such as MEO_2_MA (DEG) or MEO_3_MA (TEG). These oligomers seem to positively influence the quantum yield, leading to higher values (Figure 5b). Upon closer investigation, the enhancement of quantum yield becomes particularly remarkable when these copolymers are combined with 3-aminopropyltriethoxysilane (APTES). The introduction of APTES leads to a significant boost in the quantum yield, transforming the efficiency of ZnO QDs (Figure 5a,b). The underlying mechanism contributing to this improvement is intriguing. In this context, APTES plays a pivotal role in modifying the surface properties of the ZnO QDs. Upon addition of KOH into the medium, APTES undergoes a condensation reaction with the surface hydroxyl groups (-OH) of ZnO, resulting in the formation of a compact and uniform coating on the QD’s surface. This unique coating facilitates the attachment and integration of the copolymers, providing a stable and well-defined interface between the ZnO QDs and the polymer matrix. The reaction between the epoxy group and APTES on the surface of ZnO QDs is a crucial step in the interface modification between the QDs and copolymers. When copolymers with glycidyl (epoxy) groups are combined with ZnO QDs and APTES, a nucleophilic addition reaction occurs between the epoxy group and the amino group of APTES. This reaction is known as epoxy ring opening. This epoxy ring opening reaction results in a covalent bond between the ZnO QD and the amino group of APTES [26], ensuring a strong connection between the QD and the polymer.

**Figure 6:**
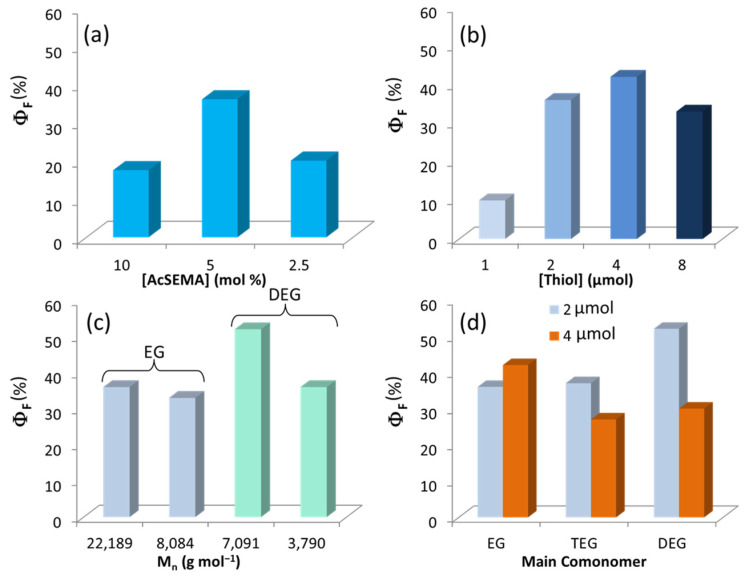
Fluorescence Quantum Yield (Φ_F_) of ZnO QDs in ethanol as a function of (**a**) AcSEMA mol (%) in the p(PEGMEMA-co-AcSEMA) copolymers; (**b**) thiol (µmol) content in the precursor solution; (**c**) Number molecular weight distribution (M_n_) for p(PEGMEMA-co-AcSEMA) (EG-AcS) and p(MEO_2_MA-co-AcSEMA) (DEG-AcS) copolymers with 5 mol % of AcSEMA; and (**d**) type of ethylene glycol based comonomer: PEGMEMA (EG), MEO_3_MA (TEG), and MEO_2_MA (DEG).

The fluorescence quantum yield of the hybrids with copolymers containing the AcSEMA linker (Figure 6) exhibits a higher performance compared to those containing GlyMA (Figure 5). As shown in Figure 6a, a thorough investigation was carried out to analyze the influence of AcSEMA content in PEGMEMA copolymers, which revealed that copolymers with an intermediate thiol content (5 mol%) exhibited an enhanced quantum yield. Moreover, it was determined that a moderate content of the thiolated polymer in the reaction solution, around 2 or 4 µmol of thiol groups, were enough to provide a robust stabilization of the ZnO quantum dots (QDs), and consequently, efficient emission (Figure 6b). Regarding the molecular weight, the study observed no significant changes in the examined weight ranges (Figure 6c). Interestingly, the type of oligoethylene glycol in the copolymer seemed to have an impact on the quantum yield, as it increased when the length of the ethylene glycol side chain in the monomer decreased (Figure 6d). This intriguing effect was also observed in cases where GlyMA was used as a comonomer. The remarkable quantum yield achieved in the AcSEMA copolymers suggests that, unlike the epoxy group, the thiol group provides adequate covalent anchoring of the polymer to the ZnO QDs. This successful anchoring mechanism is likely responsible for the elevated quantum yield observed in these copolymers.

### 3.3. Stability of ZnO QDs Hybrids in Aqueous Medium

Nowadays, fluorescent particles play a crucial role in applications ranging from biomedicine to advanced sensor manufacturing. In this regard, one key aspect to consider is their emission in water and the long-term stability. Dispersion in an aqueous medium is particularly important when it comes to biomedical applications, as often these particles need to be administered in biological systems or introduced into biocompatible fluids, as is the case of fluorescent nanoparticles used for cell tracking and visualization. To achieve this, the particles must be able to disperse uniformly in the aqueous medium to effectively interact with the target cells.

**Figure 7:**
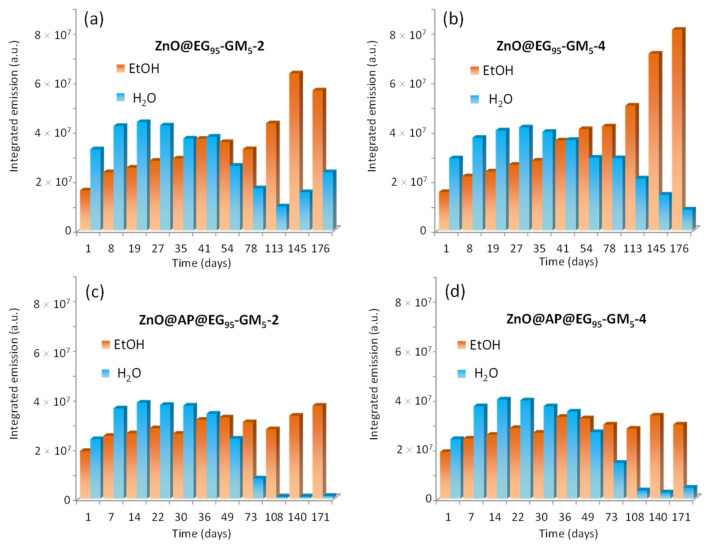
Evolution of the integrated fluorescence emission as a function of storage time for ZnO hybrids diluted 1:10 in ethanol and water synthesized in presence of (**a**) 2 µmol or (**b**) 4 µmol of GlyMA in the EG_95_-GM_5_ copolymer. Impact of APTES in combination with (**c**) 2 µmol and (**d**) 4 µmol of GlyMA in the EG_95_-GM_5_ copolymer.

**Figure 8:**
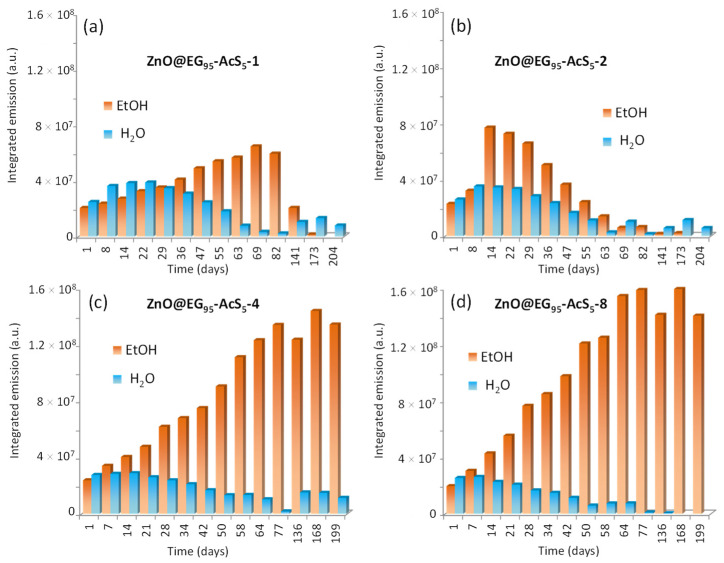
Evolution of the integrated fluorescence emission as a function of time for ZnO hybrids diluted 1:10 in ethanol and water synthesized in presence of (**a**) 1 µmol, (**b**) 2 µmol, (**c**) 4 µmol, and (**d**) 8 µmol of AcSEMA in the EG_95_-AcS_5_ thiolated copolymer.

Additionally, in environmental applications, where fluorescent particles are used as sensors to detect and quantify contaminants in water, it is essential for the particles to remain stable in the aqueous medium for extended periods of time to obtain accurate and reliable measurements. A significant finding of the present work is that the fluorescence emission of these particles does not decrease when diluted in water, but an increase in the emission is observed, particularly for ZnO QDs synthesized with copolymers bearing GlyMA functionalities (Figure 7), which is a positive characteristic for many applications. However, it has been observed that the stability in this aqueous medium is generally lower compared to ZnO QDs dispersed in ethanol (Figure 7 and Figure 8). In comparison to ethanol, where the emission remains stable for an extended period of time, at least during the 6–7 months in which it has been tested, particles in water tend to undergo a gradual decrease in fluorescence emission after about 40–50 days (Figure 7 and Figure 8). This behavior could be attributed to various factors, such as the adsorption of chemical species on the particle surface, interactions with the aqueous medium and possible aggregation formation that may affect the emission efficiency.

An intriguing observation is the significant enhancement of fluorescence emission in ZnO QDs synthesized with EG-AcS copolymers (Figure 8) over time. This enhancement seems to be linked to the polymer’s presence and becomes more pronounced with increasing polymer concentrations in the reaction medium. Interestingly, this trend is suppressed when utilizing GlyMA copolymers in combination with APTES as templates for ZnO synthesis (Figure 7c,d).

### 3.4. ZnO QDs Hybrids for Metal Detection

Previous research has demonstrated the potential of utilizing the loss of fluorescence from ZnO QDs in the presence of specific metals as sensors for detecting these analytes in water [4,5,27,28]. Specifically, a reduction in fluorescence has been observed when they are exposed to Cr^6+^, Cu^2+^, and Fe^2+^. Other authors have tested β-cyclodextrin-capped ZnO QDs as a fluorescent probe for detecting Co^2+^ [4]. Detecting metals such as copper and chromium in water is of the utmost importance due to their potential environmental and health impacts. Copper, while essential in trace amounts for human health, can become toxic at elevated levels, causing adverse effects on aquatic life and ecosystems. Chromium, especially in its hexavalent form, is a known carcinogen and possesses significant health risks to humans and animals when present in drinking water. Regular monitoring and accurate detection of these metals help ensure water quality complies with regulatory standards, safeguard public health, and enable timely remediation actions to protect the environment and prevent potential long-term consequences.

**Figure 9:**
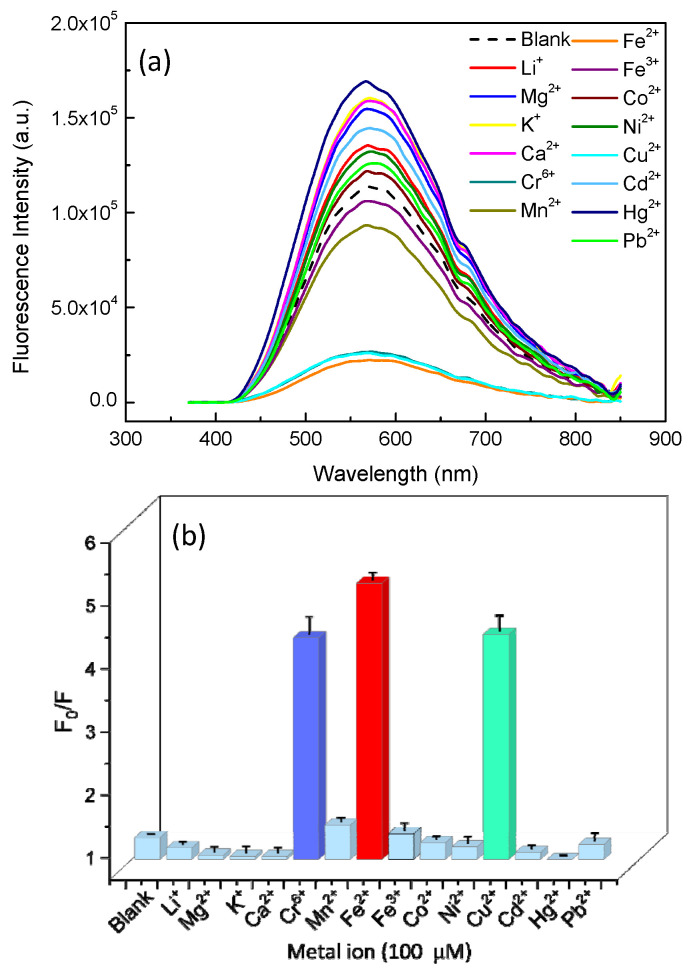
(**a**) Fluorescence intensity of ZnO@EG_95_-AcS_5_-2 QDs in the presence of aqueous solutions of several cations (Li^+^, Mg^2+^, K^+^, Ca^2+^, Cr^6+^, Mn^2+^, Fe^2+^, Fe^3+^, Co^2+^, Ni^2+^, Cu^2+^, Cd^2+^, Hg^2+^, and Pb^2+^) in a concentration of 100 μM, and (**b**) the ratio between initial integrated emission (F_0_) of the ZnO@EG_95_-AcS_5_-2 QDs and integrated emission (F) in the presence of 100 μM of the indicated metals.

In this research, we synthesized several hybrid ZnO quantum dots (QDs) to assess their potential for environmental applications. Specifically, we investigated two types of ZnO QDs nanoparticles, one coated with a thiol-bearing polymer (ZnO@EG_95_-AcS_5_-2) and the other with a polymer containing epoxy groups (ZnO@EG_95_-GM_5_-2), as “turn-off” sensors for various metal ions. Figure 9 illustrates the fluorescence emission of ZnO@EG_95_-AcS_5_-2 in the presence of various types of metal (in 100 μM aqueous solutions), highlighting the potential of nanohybrids in detecting Fe^2+^, Cr^6+^, and Cu^2+^ compared to the other metal ions we tested (Li^+^, Mg^2+^, K^+^, Ca^2+^, Mn^2+^, Fe^2+^, Co^2+^, Ni^2+^, Cd^2+^, Hg^2+^, and Pb^2+^). In Figure S2, it can be seen that the fluorescence of ZnO@EG_95_-GM_5_-2 is quenched in the presence of 100 μM solutions of Fe^2+^, Cr^6+^, and Cu^2+^, showing a very similar behavior to ZnO QDs coated with thiolated polymers (Figure 9), demonstrating that the metal detection capability resides in the interaction with the ZnO surface and it is not altered by the type of polymer on the surface.

**Figure 10:**
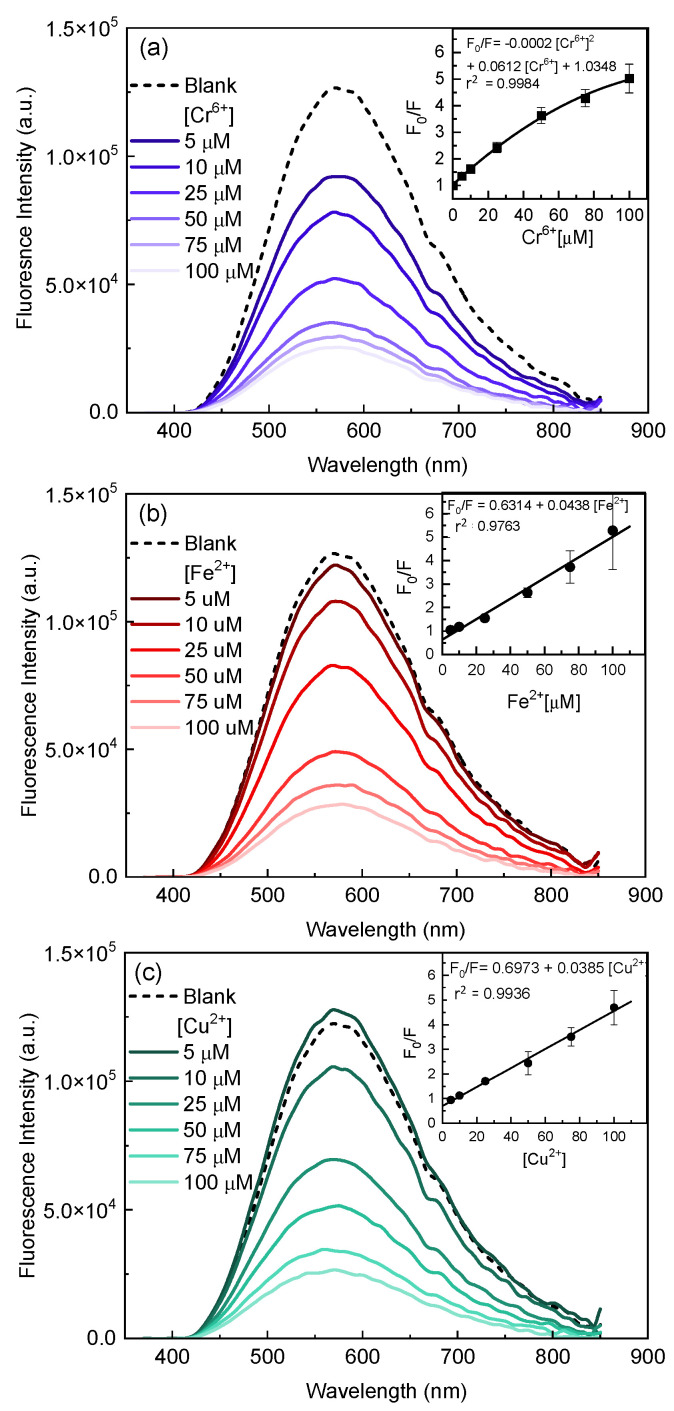
Decrease in fluorescence emission of ZnO@EG_95_-AcS_5_-2 as a function of metal concentration (5–100 μM) and in the insert the corresponding Stern–Volmer plot: (**a**) Cr^6+^; (**b**) Fe^2+^, and (**c**) Cu^2+^.

**Figure 11:**
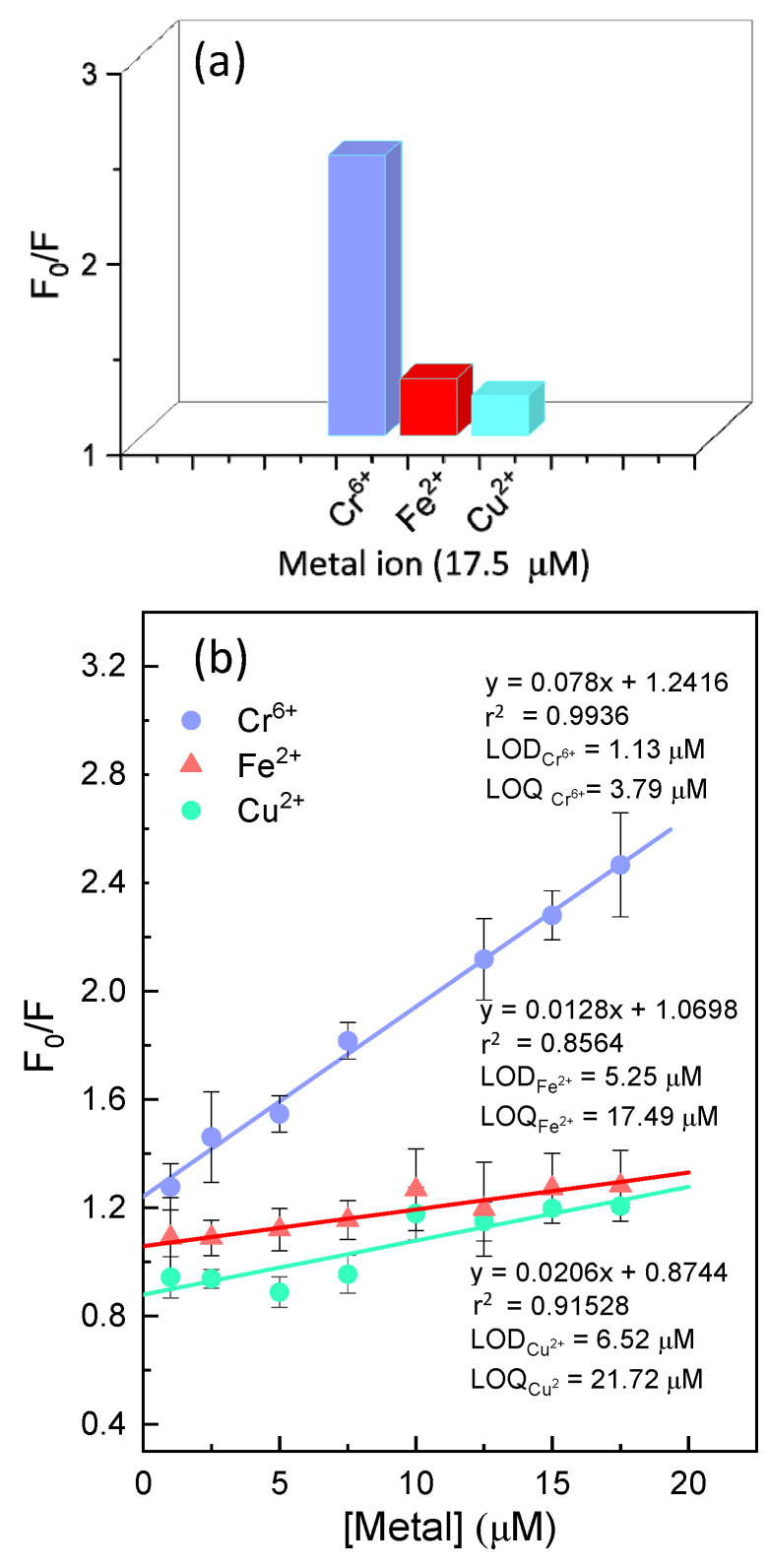
(**a**) Ratio between initial integrated emission (F_0_) of the ZnO@EG_95_-AcS_5_-2 QDs and integrated emission (F) in the presence of 17.5 μM of the indicated metals. (**b**) Stern–Volmer plot corresponding to the fluorescence emission quenching of ZnO@EG_95_-AcS_5_-2 in the concentration range of 1 to 17.5 μM.

In Figure 10, the variation in fluorescence intensity of ZnO@EG_95_-AcS_5_-2 samples in the presence of increasing concentrations (5 to 100 μM) of Cr^6+^, Fe^2+^, and Cu^2+^ is shown. In the case of Fe^2+^ and Cu^2+^, Stern–Volmer plots indicate a linear trend, while a second order dependence is observed in the case of Cr^6+^ in this concentration range. A similar behavior is observed for ZnO@EG_95_-GM_5_-2 (Figure S3). It should be noted that only 5 μM of Cr^6+^ produces a large decrease in the fluorescence intensity compared with the other two metals. For this reason, this study has also been performed with a lower concentration of the metals; these results are shown in Figure 11. Upon closer examination, at lower concentrations (1 to 17.5 μM), Cr^6^ induces significantly greater fluorescence quenching compared to Fe^2+^ and Cu^2+^. The Limit of Detection (LOD) and the Limit of Quantification (LOQ) have been determined according to LOD = 3σ/slope and LOQ = 10σ/slope (Figure 11). The LOD and LOQ values are considerably lower for Cr^6+^ in this concentration range, with the LOQ being as low as 3.79 µM and the LOD as low as 1.13 µM.

## 4. Conclusions

Well-defined copolymers of methacrylate featuring oligoethylene glycol side chains and functional groups, namely thiol and glycidyl, have been successfully synthesized via RAFT polymerization in ethanol. These copolymers serve as highly effective templates and ligands for the synthesis and protection of ZnO quantum dots (QDs). In this study, we have illustrated the inherent ability of thiol groups to interact with the surface of ZnO QDs, thereby opening new avenues for the synthesis of ZnO QDs. On the other hand, glycidyl groups provide an alternative pathway for ZnO hybrid synthesis. Under the basic reaction medium, they can either react with the hydroxyl groups on the ZnO surface or form covalent bonds with the amino groups of APTES organosilane on the ZnO QD surface. Despite a low proportion of functional groups (5% mol), both thiol-containing and glycidyl-containing polymers have been proven to be highly effective for the synthesis of hybrid ZnO QDs. Thiol-containing polymers, in particular, yield ZnO hybrids with an exceptional quantum yield (up to 52%), while polymers with glycidyl groups require the addition of APTES to achieve optimal results. Importantly, these ZnO QD hybrids exhibit s robust stability in both ethanol and aqueous environments, underscoring their potential for practical applications. This potential includes the detection of metallic ions in water, such as Fe^2+^, Cr^6+^, and Cu^2+^. Notably, the ZnO QD hybrids display remarkable sensitivity in detecting highly toxic Cr^6+^ with a Limit of Detection of 1.13 µM. Furthermore, these findings also highlight the considerable promise of these hybrid materials for bioimaging applications, provided that their toxicity is thoroughly addressed.

## Figures and Tables

**Table 1:** Summary of reaction conditions for EG-AcS, DEG-AcS, TEG-AcS, EG-GM, DEG-GM, and TEG-GM copolymers synthesis, molecular weight parameters obtained by SEC, and conversion (%) calculated by NMR analysis.

Statistical Copolymer ^a^	[M]_0_/ [CDTPA]_0_	Time (h)	M_2(theor.)_^b^ (% mol)	M_2 (exp.)_^c^ (% mol)	M_W_^d^ (g mol^−1^)	M_n_^d^ (g mol^−1^)	*Ð* ^d^	Conv. ^e^ (%)	M_n (theor.)_^f^ (g mol^−1^)
EG_90_-AcS_10_^a^	50	2	10	9	31,609	26,764	1.18	83	19,339
EG_95_-AcS_5_	50	2	5	5	25,720	22,189	1.16	81	19,716
EG_95_-AcS_5_-L	17	2	5	6	9636	8084	1.19	77	6341
EG_97.5_-AcS_2.5_	50	2	2.5	3	26,166	22,046	1.19	79	19,442
TEG_95_-AcS_5_	50	2	5	6	9823	8541	1.15	65	7512
DEG_95_-AcS_5_	50	2	5	5	9244	7091	1.30	70	6541
DEG_95_-AcS_5_-L	25	2	5	5	4866	3790	1.28	77	3623
EG_10—_DEG_85-_AcS_5_	50	2	5	4.6	13,164	10,589	1.24	74	8118
EG_80_-GM_20_	50	2	20	23	21,843	18,132	1.20	69	14,781
EG_90_-GM_10_	50	2	10	12	24,439	20,109	1.22	73	16,944
EG_95_-GM_5_	50	2	5	5	25,504	21,370	1.19	80	19,284
TEG_95_-GM_5_	50	2	5	5	9936	8395	1.18	73	8314
DEG_95_-GM_5_	50	2	5	5	9993	8673	1.15	77	7158

^a^ EG = pPEGMEMA, DEG = pMEO_2_MA, TEG = pMEO_3_MA, AcS = pAcSEMA, GM = pGlyMA. ^b^ M_2(theor.)_ = AcS, or GM theoretical content. ^c^ M_2(exp.)_ = AcS or GM determined by NMR. ^d^ *M_w_*, *M_n_* and dispersity (*Ð*) determined by SEC using a series of PMMA standards. ^e^ Conversion (%) determined by ^1^H-NMR spectroscopy. ^f^ M_n (theor.)_ = Molecular weight was calculated according to the equation: M_n (theor.)_ = ([Monomer]_0_/[CDTPA]_0_) × conversion × M_w_^Monomer^ + M_w_^CDTPA^, where M_w_^Monomer^ and M_w_^CDTPA^ are the Monomer and RAFT agent molecular weight, respectively.

**Table 2:** Synthetic conditions, hydrodynamic diameter, and photophysical properties of hybrid ZnO QDs synthesized in the presence of polymer ligands with epoxy groups and a combination of those polymers and APTES silane.

Sample	GlyMA/(µmol) ^a^	Main Monomer	GlyMA (mol %) ^b^	APTES (mol %) ^c^	Size (nm) DLS ^d^	SD (nm)	λ_max_ EtOH	Φ_F_ (%)	λ_max_ H_2_O
ZnO@EG_80_-GM_20_-2	2	PEGMEMA	20	0	5.7	0.31	556	16	568
ZnO@AP-EG_80_-GM_20_-2	2			3.5	6.0	0.41	554	40	567
ZnO@EG_90_-GM_10_-2	2		10	0	6.5	0.24	561	7	567
ZnO@AP-EG_90_-GM_10_-2	2			3.5	6.2	0.26	563	53	567
ZnO@ AP_10_-EG_90_-GM_10_-2	2			10	5.7	0.29	562	26	569
ZnO@EG_95_-GM_5_-2	2		5	0	7.0	0.43	543	11	567
ZnO@EG_95_-GM_5_-2	2			3.5	6.0	1.35	554	47	566
ZnO@EG_95_-GM_5_-4	4		5	0	5.5	0.93	551	5	568
ZnO@AP-EG_95_-GM_5_-4	4			3.5	7.1	1.16	541	38	565
ZnO@TEG_95_-GM_5_-2	2	MEO_3_MA	5	0	6.3	0.56	567	23	570
ZnO@AP-TEG_95_-GM_5_-2	2			3.5	5.9	0.34	557	33	566
ZnO@DEG_95_-GM_5_-2	2	MEO_2_MA	5	0	6.0	0.43	567	22	570
ZnO@AP-DEG_95_-GM_5_-2	2			3.5	5.9	0.34	560	32	572

^(a)^ GlyMA amount in the reaction medium. ^(b)^ GlyMA content in the copolymer. ^(c)^ APTES mol % relative to Zinc acetate. ^(d)^ Hydrodynamic size values by number distribution in ethanol estimated by DLS measurements.

**Table 3:** Synthetic conditions, hydrodynamic diameter, and photophysical properties of hybrid ZnO QDs synthesized in the presence of thiolated polymer ligands.

Sample	Thiol (µmol) ^a^	Main Monomer	AcSEMA (mol %) ^b^	Size (nm) DLS ^c^	SD (nm)	λ_max_ EtOH	Φ_F_ (%)	λ_max_ H_2_O
ZnO@EG_90_-AcS_10_-2	2	PEGMEMA	10	6.2	0.41	556	17	568
ZnO@EG_95_-AcS_5_-1	1		5	6.9	0.37	552	10	566
ZnO@EG_95_-AcS_5_-2	2		5	6.2	0.37	562	36	567
ZnO@EG_95_-AcS_5_-4	4		5	7.4	0.68	548	42	568
ZnO@EG_95_-AcS_5_-8	8		5	6.0	0.58	556	33	563
ZnO@EG_95_-AcS_5_-L-2	2		5	5.5	0.35	560	33	569
ZnO@EG_97.5_-AcS_2.5_-2	2		2.5	6.2	0.34	560	20	567
ZnO@TEG_95_-AcS_5_-2	2	MEO_3_MA	5	7.8	0.24	544	37	564
ZnO@TEG_95_-AcS_5_-4	4		5	6.2	1.31	549	30	570
ZnO@DEG_95_-AcS_5_-2	2	MEO_2_MA	5	7.8	1.5	549	52	572
ZnO@DEG_95_-AcS_5_-4	4		5	7.5	0.47	544	27	569
ZnO@DEG_95_-AcS_5_-L-2	2		5	5.8	0.42	558	36	569
EG_10—_DEG_85-_AcS_5_		PEGMEMA/MEO_2_MA	5	7.1	0.43	560	22	568

^(a)^ AcSEMA amount in the reaction medium. ^(b)^ AcSEMA content in the copolymer. ^(c)^ Hydrodynamic size values by Number distribution in ethanol estimated by DLS measurements.

## Data Availability

Data are contained within the article and Supplementary Materials.

## Supplementary Materials

The following supporting information can be downloaded at: https://www.mdpi.com/article/10.3390/polym16030429/s1. Scheme S1. Experimental procedure for the RAFT polymerization of oligoethylene glycol methacrylates with AcSEMA (a) and GlyMA (b) comonomers, using ethanol as solvent, photo-initiated by TPOL under 365 nm light and CDTPA/TPOL = 5:1. Figure S1. Representative TEM images of ZnO QDs with GlyMA copolymer and GlyMA-APTES coating: (a) ZnO@EG_95_-GM_5_-2; (b) ZnO@EG_90_-GM_10_-2; (c) ZnO@EG_80_-GM_20_-2; (d) ZnO@DEG_95_-GM_5_-2; I ZnO@TEG_95_-GM_5_-2; (f) ZnO@AP-TEG_95_-GM_5_-2. Insert show particle size histograms corresponding to the samples indicated in the Figure. Figure S2. (a) Fluorescence intensity of ZnO@EG_95_-GM_5_-2 QDs in the presence of aqueous solutions of several cations (Li^+^, Mg^2+^, K^+^, Ca^2+^, Cr^6+^, Mn^2+^, Fe^2+^, Fe^3+^, Co^2+^, Ni^2+^, Cu^2+^, Cd^2+^, Hg^2+^, and Pb^2+^) in a concentration of 100 μM and (b) ratio between initial integrated emission (F_0_) of the ZnO@EG_95_-AcS_5_-2 QDs and integrated emission (F) in the presence of 100 μM of the indicated metals. Figure S3. Decrease in fluorescence emission of ZnO@EG_95_-GM_5_-2 as a function of metal concentration (5–100 μM) and in the insert the corresponding Stern–Volmer plot: (a) Cr^6+^; (b) Fe^2+^ and (c) Cu^2+^.
