# Supplementary material for: Exploring Functional Polymers in the Synthesis of Luminescent ZnO Quantum Dots for the Detection of Cr6+, Fe2+, and Cu2+
Source: Polymers (Basel). 2024 Feb 3;16(3):429. doi: 10.3390/polym16030429 (PMC10857132; doi:10.3390/polym16030429)
Supplement: Supplementary file 1 [file polymers-16-00429-s001.zip › polymers-2851918-supplementary.pdf]

Scheme S1. Experimental procedure for the RAFT polymerization of oligoethylene glycol methacrylates with AcSEMA (a) and GlyMA (b) comonomers, using ethanol as solvent, photo-initiated by TPOL under 365 nm light and CDTPA/TPOL= 5:1……………………………….…………………..2

Figure S1. Representative TEM images of ZnO QDs with GlyMA copolymer and GlyMA-APTES coating: (a) ZnO@EG_{95}-GM_5-2; (b) ZnO@EG_{90}-GM_{10}-2; (c) ZnO@EG_{80}-GM_{20}-2; (d) ZnO@DEG_{95}-GM_5-2; (e) ZnO@TEG_{95}-GM_5-2; (f) ZnO@AP-TEG_{95}-GM_5-2. Insert show particle size histograms corresponding to the samples indicated in the Figure ……………………….…………………………………………………………………………….….2

Figure S2. (a) Fluorescence intensity of ZnO@EG_{95}-GM_5-2 QDs in the presence of aqueous solutions of several cations (Li^+, Mg^{2+}, K^+, Ca^{2+}, Cr^{6+}, Mn^{2+}, Fe^{2+}, Fe^{3+}, Co^{2+}, Ni^{2+}, Cu^{2+}, Cd^{2+}, Hg^{2+}, and Pb^{2+}) in a concentration of 100 μM and (b) ratio between initial integrated emission (F_0) of the ZnO@EG_{95}-AcS_5-2 QDs and integrated emission (F) in the presence of 100 μM of the indicated metals.………………………………………………………………………………………...………....3

Figure S3. Decrease in fluorescence emission of ZnO@EG_{95}-GM_5-2 as a function of metal concentration (5–100 μM) and in the insert the corresponding Stern–Volmer plot: (a) Cr^{6+}; (b) Fe^{2+} and (c) Cu^{2+}…..……………………………………………………………………………………………….4

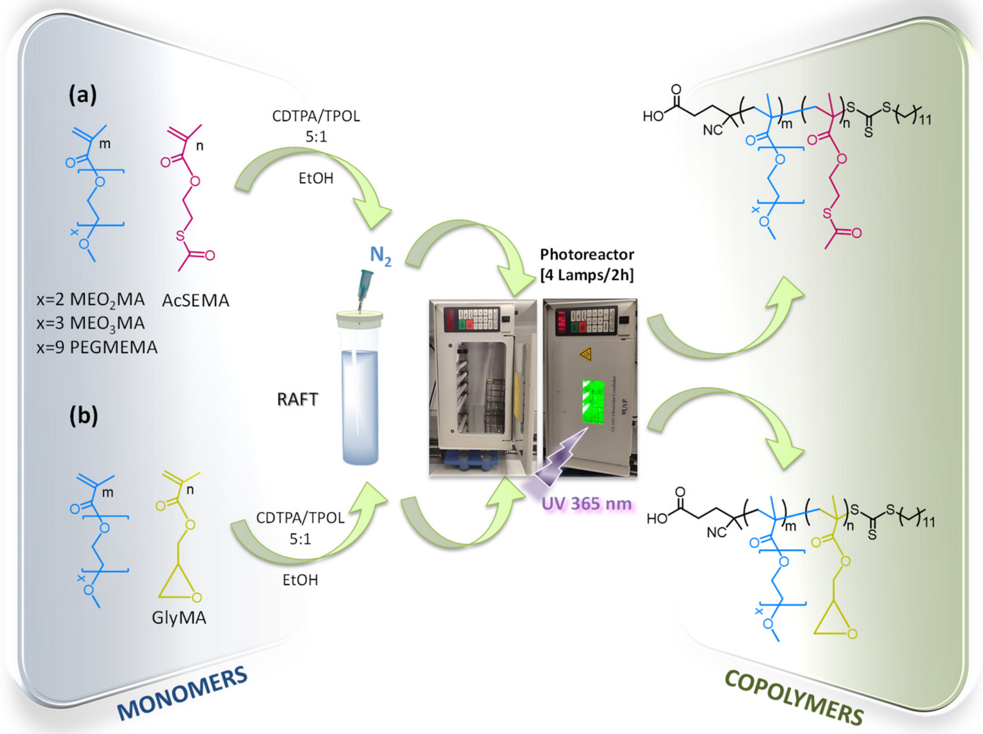

Scheme 1. Experimental procedure for the RAFT polymerization of oligoethylene glycol methacrylates with AcSEMA (a) and GlyMA (b) comonomers, using ethanol as solvent, photo-initiated by TPOL under 365 nm light and CDTPA/TPOL= 5:1.

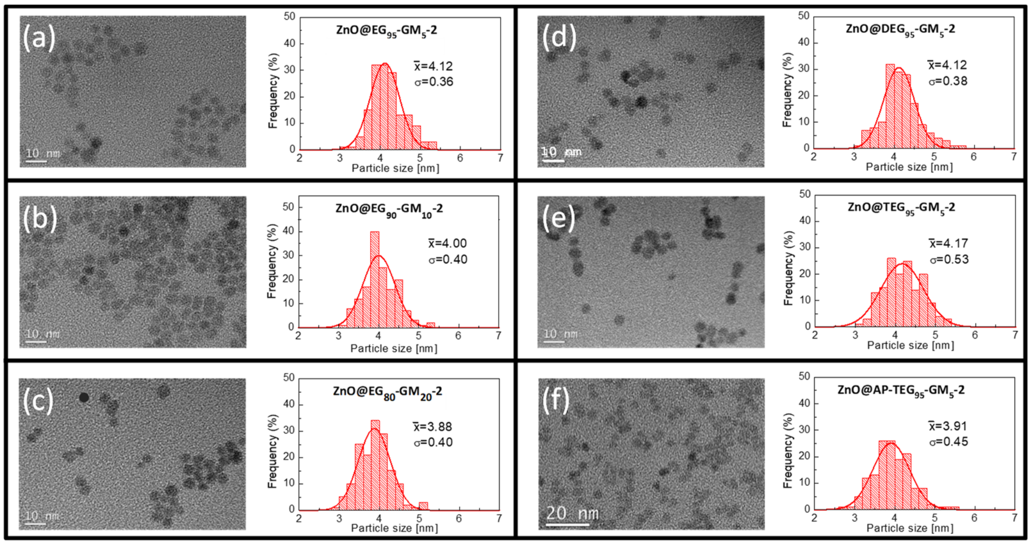

Figure S1. Representative TEM images of ZnO QDs with GlyMA copolymer and GlyMA-APTES coating: (a) ZnO@EG95-GM5-2; (b) ZnO@EG90-GM10-2; (c) ZnO@EG80-GM20-2; (d) ZnO@DEG95-GM5-2; (e) ZnO@TEG95-GM5-2; (f) ZnO@AP-TEG95-GM5-2. Insert show particle size histograms corresponding to the samples indicated in the Figure.

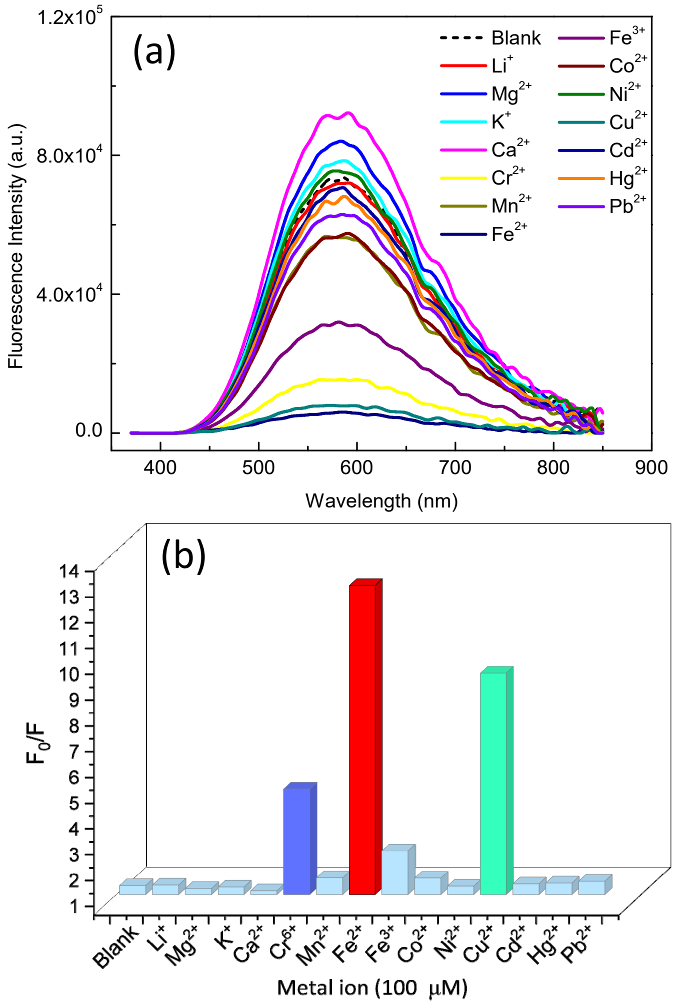

Figure S2. (a) Fluorescence intensity of ZnO@EG$_{95}$-GM$_{5}$-2 QDs in the presence of aqueous solutions of several cations (Li^{+}, Mg^{2+}, K^{+}, Ca^{2+}, Cr^{6+}, Mn^{2+}, Fe^{2+}, Fe^{3+}, Co^{2+}, Ni^{2+}, Cu^{2+}, Cd^{2+}, Hg^{2+}, and Pb^{2+}) in a concentration of 100 μM and (b) ratio between initial integrated emission (F_0) of the ZnO@EG$_{95}$-GM$_{5}$-2 QDs and integrated emission (F) in the presence of 100 μM of the indicated metals.

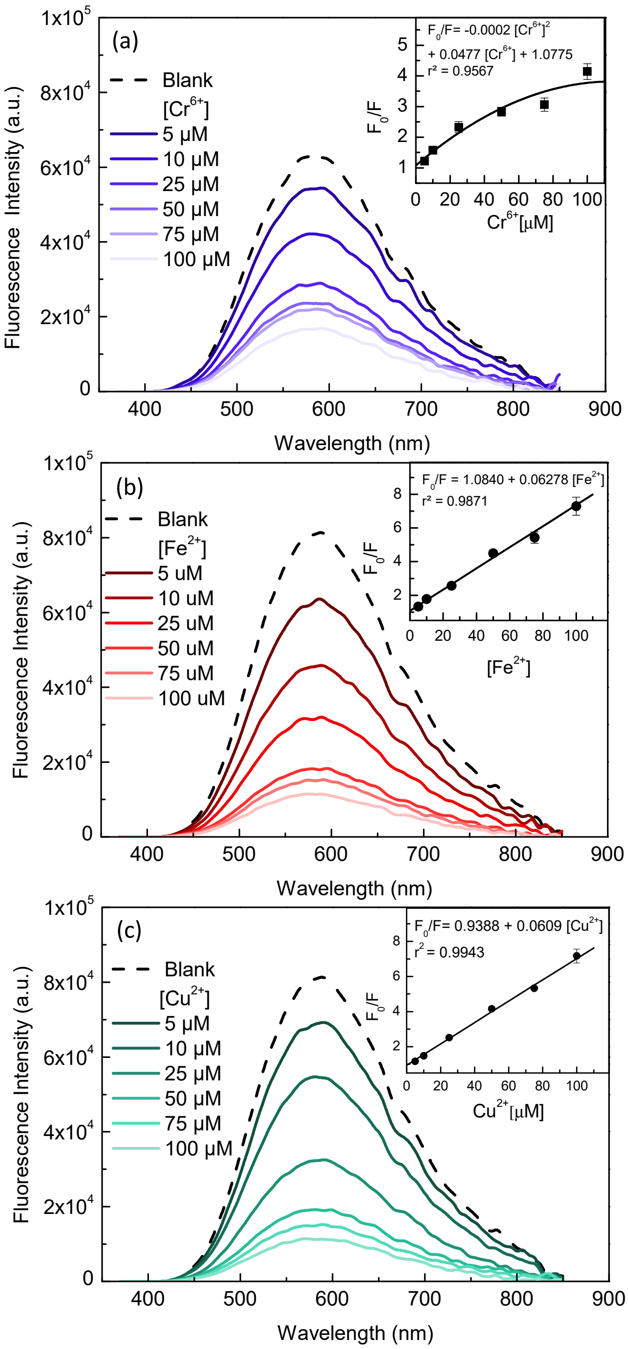

Figure S3. Decrease in fluorescence emission of ZnO@EG_{95}-GM_5-2 as a function of metal concentration (5–100 μM) and in the insert the corresponding *Stern–Volmer* plot: (a) Cr^{6+}; (b) Fe^{2+} and (c) Cu^{2+}.
